# Use of Toll-Like Receptor Agonists to Induce Ectopic Lymphoid Structures in Myasthenia Gravis Mouse Models

**DOI:** 10.3389/fimmu.2017.01029

**Published:** 2017-08-25

**Authors:** Marieke Robinet, Bérengère Villeret, Solène Maillard, Mélanie A. Cron, Sonia Berrih-Aknin, Rozen Le Panse

**Affiliations:** ^1^INSERM U974, Paris, France; ^2^UPMC Sorbonne Universités, Paris, France; ^3^AIM, Institut de myologie, Paris, France

**Keywords:** toll-like receptor, lipopolysaccharide, poly(I:C), autoimmunity, B cells, myasthenia gravis, germinal centers

## Abstract

Myasthenia gravis (MG) is an autoimmune disease mediated by autoantibodies against the acetylcholine receptor (AChR) at the neuromuscular junction. MG symptoms are characterized by muscle weaknesses. The thymus of MG patients is very often abnormal and possesses all the characteristics of tertiary lymphoid organs such as neoangiogenesis processes, overexpression of inflammatory cytokines and chemokines, and infiltration of B lymphocytes leading to ectopic germinal center (GC) development. We previously demonstrated that injections of mice with polyinosinic–polycytidylic acid [Poly(I:C)], a synthetic double-stranded RNA mimicking viral infection, induce thymic changes and trigger MG symptoms. Upon Poly(I:C) injections, we observed increased thymic expressions of α-AChR, interferon-β and chemokines such as CXCL13 and CCL21 leading to B-cell recruitment. However, these changes were only transient. In order to develop an experimental MG model associated with thymic GCs, we used Poly(I:C) in the classical experimental autoimmune MG model induced by immunizations with purified AChR emulsified in complete Freund’s adjuvant. We observed that Poly(I:C) strongly favored the development of MG as almost all mice displayed MG symptoms. Nevertheless, we did not observe any ectopic GC development. We next challenged mice with Poly(I:C) together with other toll-like receptor (TLR) agonists known to be involved in GC development and that are overexpressed in MG thymuses. Imiquimod and CpG oligodeoxynucleotides that activate TLR7 and TLR9, respectively, did not induce thymic changes. In contrast, lipopolysaccharide that activates TLR4 potentiated Poly(I:C) effects and induced a significant expression of CXCL13 mRNA in the thymus associated with a higher recruitment of B cells that induced over time thymic B-lymphoid structures. Altogether, these data suggest that tertiary lymphoid genesis in MG thymus could result from a combined activation of TLR signaling pathways.

## Introduction

Secondary lymphoid organs (SLOs) such as lymph nodes, spleen, or tonsils are environment specialized for the interaction between lymphocytes and antigen-presenting cells in order to develop an adaptive immune response. SLOs provide the optimized microenvironment for germinal center (GC) reactions. When T and B cells enter into SLOs *via* the vascular network, they can encounter primed antigen-presenting cells engaging a GC reaction. Within GCs, B cells undergo clonal expansion, immunoglobulin (Ig) class switch, and somatic hypermutation leading to the development of B cells expressing high-affinity antibodies that differentiate into antibody-secreting plasma cells and memory B cells in order to mediate a sustained protection against invading pathogens. Chronic inflamed tissues can turn into tertiary lymphoid organs (TLOs) associated with ectopic GC reactions. These tissues are characterized by the development of a vascular system, the infiltration of leukocytes, the presence of GCs, sustained by the overexpression of chemokines and inflammatory cytokines (1). TLOs are observed in many organ-specific autoimmune diseases such as in the thymus in myasthenia gravis (MG), the salivary glands in Sjogren’s syndrome, the thyroid gland in Graves’ disease and Hashimoto’s thyroiditis, and the cerebral meninges in multiple sclerosis (2, 3). The common feature for all these diseases is that tertiary lymphoid neogenesis occurs in tissues harboring the autoantigen.

Myasthenia gravis with anti-acetylcholine receptor (AChR) antibodies is characterized by muscle weakness and fatigability. MG is a prototype autoimmune disease in which the target organ, the muscle, is distinct from the effector organ, the thymus. In MG patients with anti-AChR antibodies, functional and morphological abnormalities of the thymus are frequently observed: either a thymoma or B-cell infiltrations with more than 75% of patients exhibiting thymic hyperplasia of lymphoproliferative origin with ectopic GC development (4). There is a clear relationship between the degree of hyperplasia and the serum level of anti-AChR antibodies (4), and a recent randomized clinical trial has clearly demonstrated that thymectomy improved clinical outcomes (5). Moreover, immunodeficient mice engrafted with human MG thymic tissue have anti-AChR antibodies in the serum and animals displayed MG-like symptoms that correlated with the loss of AChR at the muscle endplates (6). Altogether these data demonstrate that the thymus is clearly involved in the MG. The hyperplastic MG thymus displays all the characteristics of TLOs (7): neoangiogenic processes with high endothelial venules (HEVs) and lymphatic vessels development (2, 8, 9), chemokine overexpression (such as CXCL13 and CCL21) favoring peripheral-cell recruitment (8, 10, 11), and ectopic GC development (4). Moreover, the autoantigen (α-AChR) involved in MG is directly expressed in the thymus by thymic epithelial cells (TECs) and myoid cells (12). Within thymic GCs, interactions have been described between T follicular helper cells and B cells known to induce B-cell maturation and antibody production in the SLOs (13). The presence of anti-AChR autoreactive T cells (14) and B cells producing anti-AChR antibodies (15, 16) has also been described in the thymus of MG patients. The exact mechanisms initiating these thymic changes and the intrathymic autoimmune response to AChR are not yet clearly defined but local inflammation seems to be mandatory. The overexpression of interferon (IFN)-β and IFN-I-induced genes has been observed in the MG thymus even long after the disease onset (17, 18). Later on, our team demonstrated that IFN-β could be the orchestrator of thymic changes associated with MG. Indeed, IFN-β induces specifically α-AChR expression in TECs. IFN-β also increases TEC death and the uptake of TEC proteins by dendritic cells, suggesting a role in α-AChR sensitization. In parallel, IFN-β increases the expression of the chemokines CXCL13 and CCL21 by TECs and lymphatic endothelial cells, respectively. These two chemokines are involved in GC development and are overexpressed in MG thymus with follicular hyperplasia. We also demonstrated that the B-cell activating factor, which favors autoreactive B-cells, was overexpressed by TECs in MG thymus and was induced by IFN-β in TEC cultures (19).

Myasthenia gravis, as other autoimmune diseases, is a multifactorial disease involving genetic susceptibility, hormonal influence (20) or environmental factors including pathogen infections (21). Persistent viral infections in specific organ are suspected to favor tertiary lymphoid genesis associated with autoimmunity (22). The thymus of MG patients is characterized by increased expression of toll-like receptors (TLRs) involved in pathogen recognition, such as TLR3, TLR4, TLR7, and TLR9, but also by increased expression of IFN-β and numerous IFN-induced genes (17, 18, 23–26). All these data suggest that MG could be triggered by pathogen infections.

A well-defined experimental autoimmune MG (EAMG) model is induced by immunizing wild-type mice with a mixture of complete Freund’s adjuvant (CFA) and AChR purified from *Torpedo californica* fish (27). In this model, mice develop antibodies against T-AChR which target AChR on muscle cells. Even though the EAMG model is relevant to study muscle weakness caused by the anti-AChR antibody attack, it does not completely recapitulate the human disease, as the thymus does not display ectopic GC development (28). Using a transgenic mouse line with thymic overexpression of CXCL13, we clearly demonstrated that inflammation is mandatory to induce B-cell recruitment and GC development in the EAMG model (29). Using polyinosine–polycytidylic acid [Poly(I:C)], a synthetic analog of double-stranded (ds)RNA mimicking viral infection, we demonstrated that repeated injections of C57BL/6 mice rapidly induce thymic changes similar to what could happen in MG patients. Briefly, after 1 week of injections, we observed thymic induction of IFN-β associated with an overexpression of CXCL13 and CCL21, leading to transient thymic B-cell recruitment. In parallel, we also demonstrated the specific overexpression of α-AChR leading after several weeks of Poly(I:C) injections to an anti-AChR autoimmune response characterized by the production of anti-AChR antibodies, a specific proliferation of B cells and MG-like clinical signs. However, in this MG mouse model we do not observe the development of thymic GCs. In fact, thymic changes are transiently observed after 1 week of injections and disappear thereafter (18, 19).

The aim of the study was to find a combination of triggers to better recapitulate the human MG disease associated with thymic pathology including GC development. Compared to other TLR agonists, such as those activating TLR4, TLR7 and TLR9, Poly(I:C) is not well known to favor the development of GCs as fully reviewed in Table 1. We then hypothesized that the combined activation of Poly(I:C) signaling pathway in the classical EAMG model or the combined use of Poly(I:C) with other TLR agonists could sustain thymic changes and favor the effective development of thymic GCs as observed in MG patients.

**Table 1:** Implication of toll-like receptor (TLR) signaling pathways in germinal center (GC) development in mice.

	TLR implication in GC development in mice
TLR1	TLR1 is expressed mainly on follicular-B cells compared to other naive-B cells (36).
TLR3	TLR3 ligand enhances GC formation in the spleen during the tetanus toxoid vaccine response (35).
TLR4	TLR4 is expressed on follicular-B cells and other naive-B cells (36). TLR4-mediated activation of B cells may help to feed and stabilize spontaneous and ectopic GCs (57). TLR4 signaling by follicular dendritic cells is central for GC onset (58).
TLR7	TLR7 is expressed on follicular-B cells and other naïve-B cells (36). TLR7 exerts B-cell intrinsic effects in promoting spontaneous GC and plasmablast B-cell development (39). Using TLR7 knockout mice, an essential role of TLR7 in spontaneous GC development is observed (40). TLR7 is essential for spontaneous GC response including B-cell expansion and diversification in lupus-prone mice (41). GC B cells in TLR7-deficient mice proliferated to a lesser extent (42). Extra copy of TLR7 (on the Yaa locus) relaxes the stringency for selection in the GCs resulting in increased autoreactivity of the antigen-driven B-cell repertoire (43). TLR7 ligand within virus-like particles largely restores defective GC reaction and antibody responses in IL-21R-deficient mice (44). TLR7 activation in follicular dendritic cells promotes autoreactive B-cell responses (45).
TLR9	TLR9 is expressed on follicular-B cells and other naïve-B cells (36). CpG is one of the most potent B-cell mitogens known (46). CpG DNA markedly augments plasma-B cell generation from GC-B cells (47). TLR9 signaling in dendritic cells and B cells controls the magnitude and quality of the GC response, respectively (48).

## Materials and Methods

All reagents were purchased from Sigma-Aldrich (Saint Quentin Fallavier, France) unless indicated otherwise.

### Animals

C57BL/6 female mice were purchased from Janvier Labs (Saint-Berthevin, France) and housed 1–2 weeks before experiments in a SPF animal care facility (CEF – Pierre and Marie Curie University, Paris, France). The study was approved by the local Ethics Committee (agreement no 2569.01).

### TLR Agonist Injections in Mice

6- to 8-week-old C57BL/6 mice were injected (i.p.) with Poly(I:C) 200 µg (tlrl-pic-5, Invivogen, Toulouse, France), Imiquimod 20 µg (tlrl-imqs, Invivogen), CpG (class C) oligodeoxynucleotides 25 µg (ODN 2395, Invivogen), lipopolysaccharide (LPS) 10 µg (ALX-581-007, Invivogen: low doses of LPS were used to avoid endotoxemia), or physiological water. For short-term experiments, mice were injected three times every other day and sacrificed the day after the last injection. For long-term experiments, mice were injected with TLR agonists every three days throughout the experiment.

### *Torpedo californica* AChR (T-AChR) Extraction and Purification

250 g of frozen electric organ tissue from *T. Californica* (EastCoast Bio, North Berwick, ME, USA) were minced and homogenized at 4°C in few milliliters of buffer A [0.01 M Tris, 1 mM ethylenediaminetetraacetic acid (EDTA), 0.5 mM sodium azide (NaN_3_), 0.1 mM phenylmethylsulfonyl fluoride (PMSF), 0.1 M NaCl]. The filtrate was centrifuged at 8,000 rpm for 30 min at 4°C. The pellet was resuspended in 400 mL of buffer B (0.01 M Tris, 1 mM EDTA, 0.5 mM NaN_3_, and 0.1 mM PMSF) and centrifuged as above. The pellet was resuspended in a total of 8 mL of Buffer B supplemented with Triton X-100 and NaCl to a final concentration of 1% and 0.1 M, respectively. After stirring for 1 h at room temperature the suspension was centrifuged at 38,000 rpm for 1 h at 4°C. The supernatant was mixed with alpha-cobratoxin (L8114-1MG, Latoxan, Portes les valence, France) coupled to sepharose beads (as described below) and gently stirred overnight at 4°C for adsorption. The mix was filtered on a sinter glass once with 100 mL of Buffer A containing 0.1% Triton X-100, once with Buffer B containing 1 M NaCl and finally again with Buffer A containing 0.1% Triton X-100. The elution was performed by stirring the beads for 1–2 h with 10 mL of elution buffer made of 0.7 M carbamyl choline chloride (C-4382) in dialysis buffer. Eluent was then filtered on a sinter glass as described above. The purified receptor was dialyzed for 15 h at 4°C in 1–2 L of dialysis buffer (0.01 M Tris, 1 mM EDTA, 0.5 mM NaN_3_, 0.1% Triton X-100, and 0.1 M NaCl). The receptor quantification was then performed by BCA assay and stored at −80°C until used.

Alpha-cobratoxin coupled to sepharose beads were prepared as followed. Sepharose beads (4 g) were mixed 15 min at room temperature with 40–50 mL of 1 mM HCl and filtered on a sinter glass with 100 mL of 1 mM HCl. Beads were washed with coupling buffer (0.1 M NaHCO_3_, 0.5 M NaCl), added to 15 mg of alpha-cobratoxin pounder dissolved in coupling buffer, and incubated for 2 h at room temperature. After filtration, the remaining active groups were blocked by adding about 100 mL of 0.2 M glycine and stirred 30 min at room temperature. The beads were washed with buffer A before used.

### Experimental Autoimmune Myasthenia Gravis (EAMG)

Experimental autoimmune MG model was done mainly as described by Tuzun et al. with slight modifications (27). 6–8 week-old C57BL/6 mice were immunized with T-AChR. T-AChR was emulsified with an equal volume of CFA (F5881) supplemented with *Mycobacterium tuberculosis* (MTB) 10 mg/mL (H37RA, BD Difco, Villepinte, France). Mice were subcutaneously injected (200 µL/mouse, 30 µg AChR) at several sites (hind foot-pads, tail base, and in the back). After 4 weeks, mice were immunized a second time with T-AChR and CFA emulsion. A third immunization was done if the percentage of sick mice was less than 50%. Mice were euthanized 2–3 weeks after the last immunization for assessment of immunopathological parameters. Control mice were injected similarly with CFA emulsion devoid of T-AChR. Mice were regularly monitored for signs of muscle weakness, and mice that were too weak as defined by the ethical committee were euthanized.

### Clinical Evaluation of Mice

Different assessments were taken into account to evaluate the clinical state of the animals. Mice were weighed every week. Muscle strength was analyzed by measuring the forelimb strength with a grip strength apparatus. As clinical signs are not always obvious in resting mice, the grip test measurements were done after a 3-min run on a treadmill. In experiments on the EAMG models, an inverted grid test was also carried out. Mice were tired by gently dragging them 20 times across the top grid of a cage and then carefully observed as the grid was rotated. The time at which the mouse falls-off, the behavior of mice is noted. Individually, these tests are not always powerful enough to determine if a mouse was sick. However, the combination of the different tests allows establishment of a global clinical score that is much more relevant. A global clinical score was then calculated as previously described taking into account the loss of weight, the grip test, and the grid test results for T-AChR-immunized mice compared to control mice. A mouse was considered sick when it reached a global clinical score of 2 (29). In long-term experiments using only TLR agonist injections, the clinical evaluations were done regularly. For the global clinical score evaluation, a rotarod test was done instead of the inverted grid test and a mouse was considered sick with a global clinical score above 3. In the course of the experiments, mice that were considered too sick were euthanized and classified with the higher global clinical score.

### ELISA

To detect anti-AChR antibodies, 96-well ELISA plates were coated overnight at 4°C with 0.5 µg/mL of T-AChR diluted in 10 mM NaHCO3 buffer, pH 9.6. T-AChR-coated wells were blocked with 10% SVF in PBS at 37°C for 2–3 h. 100 µL of mouse serum (1:100,000 for EAMG experiments or 1/100 for other experiments) or 5 µg of thymic extracts were used per well and incubated for 90 min at 37°C. Subsequently, wells were washed four times with a PBS-0.05% Tween buffer. 100 µL of 1/10,000 diluted biotinylated polyclonal anti-mouse IgGs (E0413, Dako, Courtaboeuf, France) were added for 90 min at 37°C. Next, samples were incubated with 100 µL of streptavidin–horseradish peroxidase 1:10,000 (PN IM0309, Beckman Coulter, Villepinte, France).

To measure the expression level of all IgG, 96-well plates were coated overnight at 4°C with a polyclonal goat anti-mouse IgGs (1 µg/mL) (Z0420, Dako). After blocking for 1 h with PBS-BSA 2%, mouse serum (1:100,000) or standards (Clone DAK-GO1, X0931, Dako) were incubated for 90 min at 37°C and, subsequently, 1:10,000 diluted biotinylated anti-mouse IgGs and 1:10,000 streptavidin–horseradish peroxidase were added.

Tetramethylbenzidine was used for color development, and the optical density at 450 nm was measured with a microtiter plate reader spectrophotometer. The levels of anti-AChR antibodies were normalized on the total level of IgG.

### Flow Cytometry

Single cell suspensions from thymus were prepared by passing the organs through a nylon mesh. Isolated cells were incubated for 30 min on ice with antibodies from BD Bioscience, except when specified: anti-CD19 PE (553786, clone 1D3 BD) or CD19-efluor450 (48-0193-82, clone eBio1D3, eBioscience, Paris, France), anti-CD4 Alexa700 (557956, clone RM4-5 BD) or anti-CD4-APC (553051, clone RM4-5 BD), anti-CD8a PE-Cy7 (552877, clone 53-6.7 BD). Flow cytometry was performed on a FACS Verse (BD Biosciences) and data analyzed using FlowJo software.

### Quantitative RT-PCR

Total RNA was extracted in TRIzol (Invitrogen) using the FastPrep FP120 instrument (Qbiogen, Illkirch, France). RNA (1 µg) was reverse-transcribed for 1 h at 42°C using AMV (Ref 10109118001, Roche Life Science, Meylan, France) with oligo-dT (Invitrogen). RT-PCR reactions were performed with the LightCycler^®^ 480 System (Roche). The primer sequences (Eurogentec, Angers, France) are listed in Table S1 in Supplementary Material. All samples were normalized to GAPDH.

### Immunohistochemistry

Cryosections of thymic samples (7 µm) were fixed in ice-cold acetone for 20 min and unspecific binding sites blocked with 2% BSA. Sections were stained with anti-K5-FITC polyclonal antibody (PRB-160P, Eurogentec) for medullary TECs, while B cells were detected with a biotinylated anti-B220 antibody (clone RA3-6B2, BD bioscience) and streptavidin Alexa-Fluor-594 (S11227, Invitrogen). Images were acquired with a ZeissAxio Observer Z1 Inverted Microscope. The number of B cells was counted in 6–8 fields representative of thymic sections.

### Statistical Analyses

For 2-by-2 comparisons, non-parametric Mann–Whitney test was applied as specified in figure legends.

## Results

### Effects of Poly(I:C) Injections on the Classical EAMG Model

In order to obtain a EAMG model associated with tertiary lymphoid genesis, we investigated the combined effects of Poly(I:C) injections together with the classical immunization protocol used to induce EAMG.

**Figure 1:**
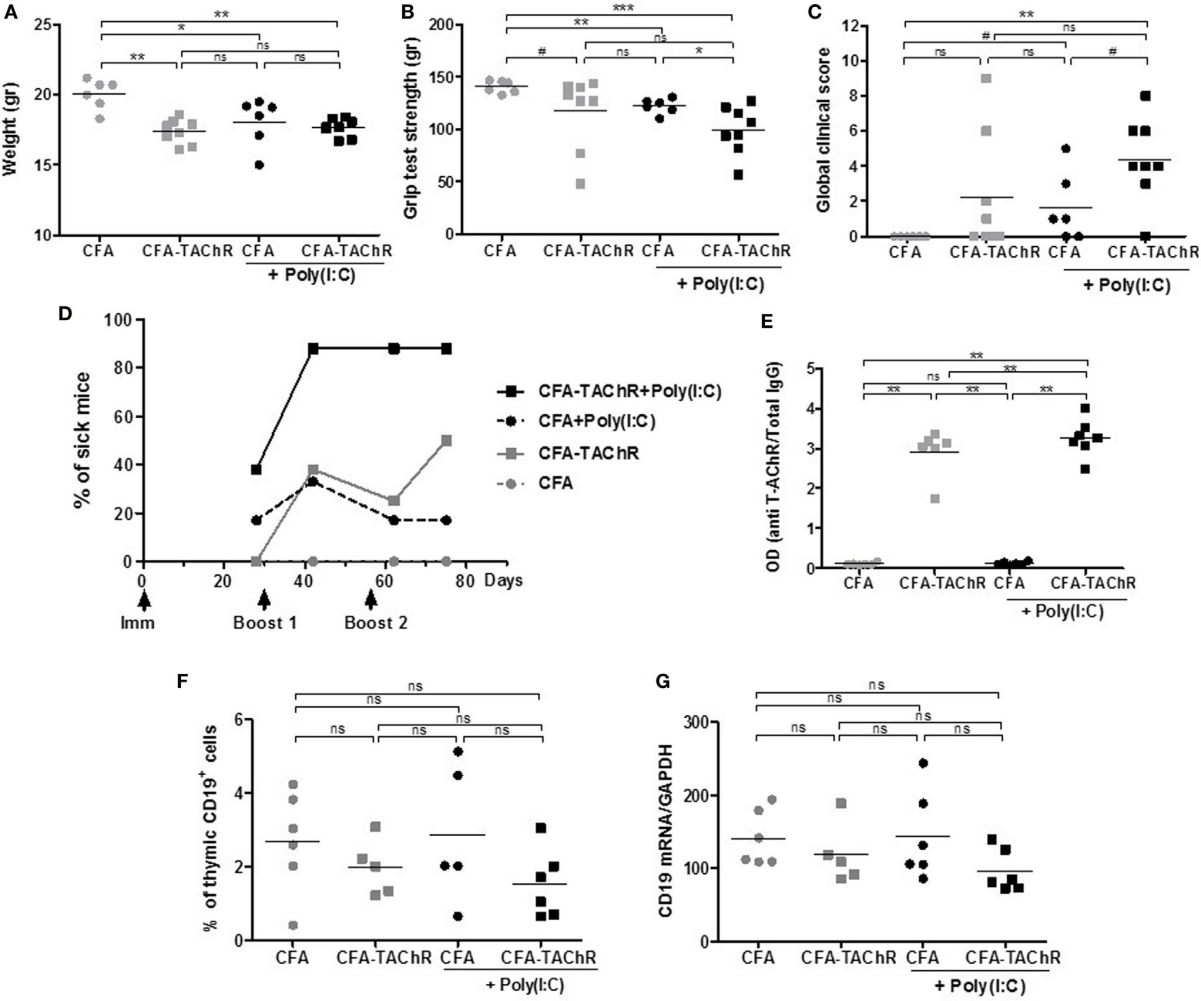
Effects of polyinosinic-polycytidylic acid [Poly(I:C)] injections on the classical experimental autoimmune myasthenia gravis (EAMG) model. C57BL6 mice were immunized at day 0 and boosted twice at days 30 and 56 with complete Freund’s adjuvant (CFA) or CFA/*Torpedo californica* AChR (T-AChR). i.p. injections of Poly(I:C) were done twice a week for the groups CFA/Poly(I:C) and CFA/T-AChR/Poly(I:C). Clinical evaluations were done at days 28, 42, 62, and 75, and data obtained at day 42 are shown on graphs A–C. **(A)** Mice were weighted. **(B)** Muscle strength was measured with the grip test apparatus after exercise on a treadmill. **(C)** A global clinical score for each mouse was calculated taking into account the weight loss, the grip test, and the inverted grid test. **(D)** The percentages of mice with a global clinical score of at least 2 and clear muscle weakness symptoms are shown in kinetic and statistical analyses were done to compare T-AChR groups to their respective CFA control groups. **(E)** ELISA for anti-AChR antibodies in serum taken 2 weeks after the last immunization. Data were normalized on the total level of IgGs. **(F)** Thymuses were analyzed by flow cytometry for the percentage of B cells (CD19^+^ cells) or **(G)** by PCR for the level of CD19 mRNA expression. *p*-Values were assessed by the Mann–Whitney test and annotated as follow on graphs: ^#^<0.1, *p < 0.05, **p < 0.01, ***p < 0.001.

Two independent experiments were carried out and representative results are given in Figure 1. Mice immunized with T-AChR according to the classical EAMG protocol (CFA/T-AChR), displayed weight loss (Figure 1A), a decreased grip strength (Figure 1B) and taking into account the global clinical score, 50% of the animals were considered sick at the end of the experiment (Figures 1C,D). It is important to note that mice are quite resistant to the EAMG model and not all treated animals get sick (27). Mice immunized with T-AChR in the presence of Poly(I:C) [CFA/TAChR/Poly(I:C)] displayed a worsening of clinical signs (Figures 1C,D). Using the global clinical score, we detected that seven out of eight animals (87.5%) were already sick after the first boost (Figures 1C,D; Figure S1A in Supplementary Material).

Although animals were clearly more affected when Poly(I:C) was injected, the levels of circulating anti-AChR measured were not really increased compared to animals immunized only with CFA/T-AChR (Figure 1E; Figure S1B in Supplementary Material). In the EAMG model, mice develop first large amounts of antibodies against the T-AChR and afterward autoantibodies against the mouse AChR that are probably more pathogenic (30). In the EAMG model, our ELISA test did not discriminate between mouse AChR and T-AChR autoantibodies but higher level of pathogenic mouse AChR antibodies upon Poly(I:C) injections could explain the increased MG symptoms.

In the control group corresponding to mice injected with CFA/Poly(I:C), we observed an induction of MG clinical signs with a comparable decrease in weight and grip strength and increase in the global clinical score compared to CFA/T-AChR mice (Figures 1A–D). As previously observed, Poly(I:C) is able to induce by itself the production of mouse AChR autoantibodies that can be detected by radioimmunoassay (18) and with our ELISA tests (data not shown). However, the levels of anti-mouse AChR antibodies induced by Poly(I:C) were 100–200 less than those produced by CFA/T-AChR injections and not detectable in the present ELISA tests in which serum was diluted at 1:100,000 (Figure 1E).

As Poly(I:C) worsened the MG symptoms in EAMG model, we analyzed the thymus of the animals at the end of the experiments. By flow cytometry, we did not detect more thymic B cells in the different subgroups (Figure 1F). By PCR, we did not observe either an increase in CD19 expression (Figure 1G).

Altogether these results demonstrated that Poly(I:C) injections greatly favor MG symptoms in the classical EAMG model as almost all animals were already sick after the first boost. However, we did not observe thymic changes at the end of the experiments suggesting that specific signal(s) was (were) missing to induce ectopic GC development as in the human MG disease. As Poly(I:C) is not considered to be involved in GC development, we investigated the effects of other TLR agonists (Table 1).

### Effects of Imiquimod and CpG Together with Poly(I:C) Injections

**Figure 2:**
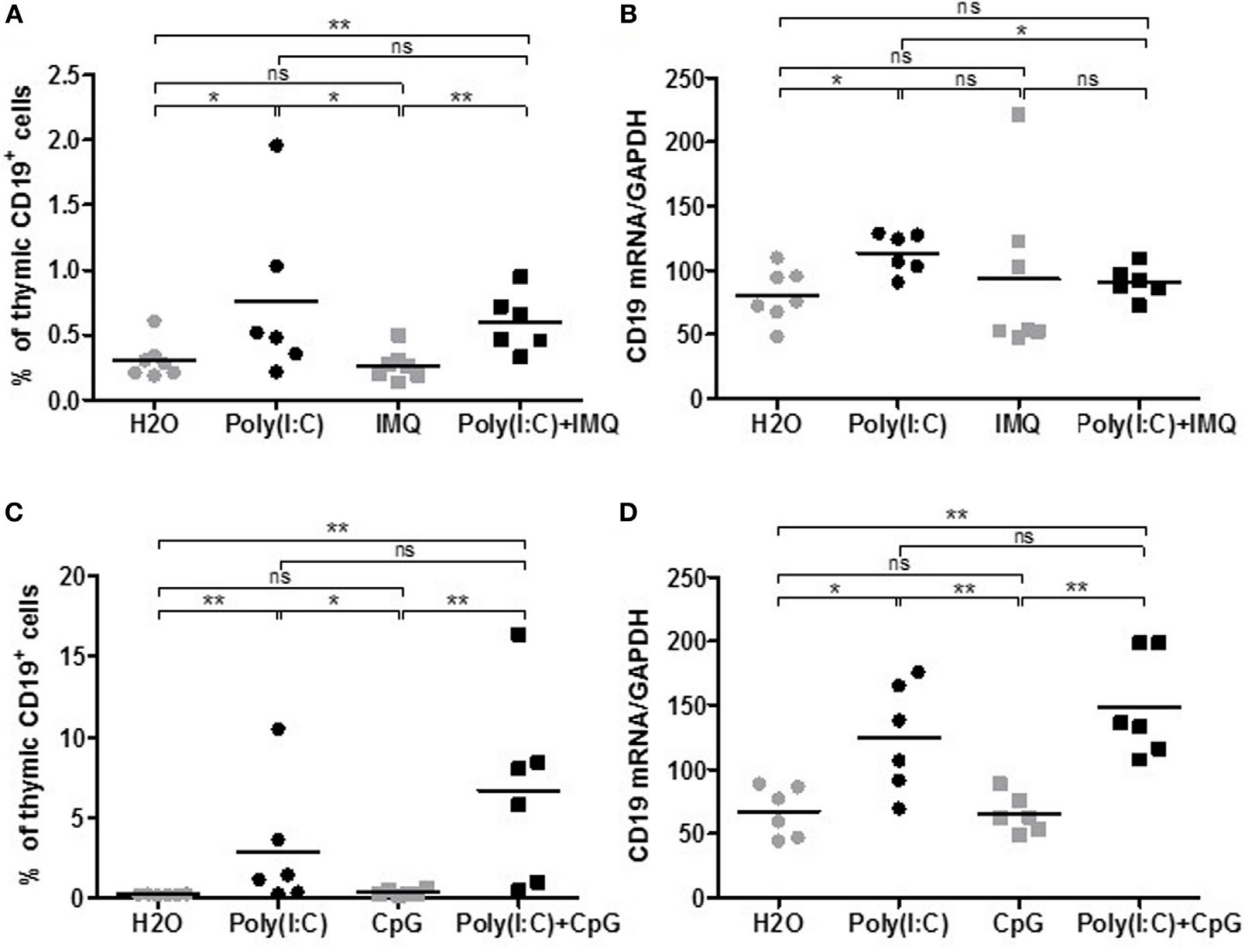
Effects of Imiquimod, CpG-C with polyinosinic-polycytidylic acid [Poly(I:C)] on thymic B cells. C57BL6 mice (*n* = 6–7 per group) were i.p. injected every 2 days for 1 week with physiological water (control), Poly(I:C) 200 µg, Imiquimod (IMQ) 20 µg, Poly(I:C) + IMQ, CpG-C 25 µg, or Poly(I:C) + CpG. Thymuses were analyzed by flow cytometry for the percentage of B cells (CD19^+^ cells) in mice injected with IMQ **(A)** or with CpG-C **(C)**. Thymuses were analyzed by PCR for CD19 mRNA, and data were normalized on GAPDH for mice injected with IMG **(B)** or CpG-C **(D)**. *p*-Values were assessed by the Mann–Whitney test and annotated as follow on graphs: *p < 0.05, **p < 0.01, ***p < 0.001.

Polyinosinic–polycytidylic acid injections in mice are able to induce thymic changes after 1 week and MG symptoms associated with anti-AChR production after several weeks. However, we do not observe persistent thymic changes (18). We then investigated if we could exacerbate or sustain Poly(I:C) effects with simultaneous injections of agonists of other TLR, such as TLR7 and TLR9, that could be involved in GC formation (Table 1). TLR7 is activated by ssRNAs from viral infections. We used Imiquimod (Figures 2A,B), an imidazoquinoline amine analog to guanosine that specifically activates TLR7 but not TLR8. For TLR9 activation (Figures 2C,D), we used CpG oligodeoxynucleotides that correspond to short synthetic single-stranded DNA molecules containing unmethylated CpG dinucleotides mimicking bacterial DNA. We selected CpG class C that combines features of CpG class A favoring IFN-α production from plasmacytoid dendritic cells, and CpG class B inducing B-cell proliferation.

After 1 week of injections, analyzing the percentage of thymic B cells by flow cytometry (Figures 2A–C) or the expression by PCR of the B-cell marker CD19 (Figures 2B–D), we did not observe any effects of Imiquimod or CpG-C by themselves on the recruitment of thymic B cells. We did not either observe any additional effects when used with Poly(I:C). The same observations were made on CXCL13, CCL21, IFN-β, or α-AChR mRNA expression (data not shown). Consequently, we did not investigate further the effect of Imiquimod and CpG-C.

### Effects of LPS Together with Poly(I:C) Injections

**Figure 3:**
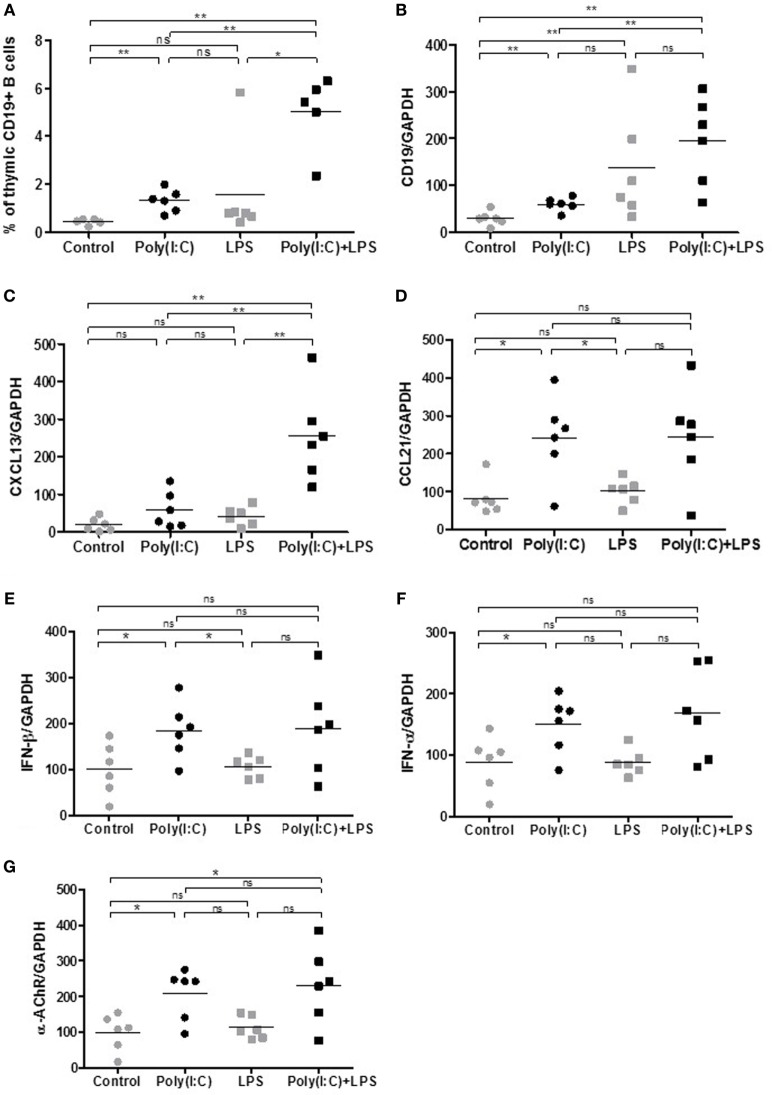
Thymic changes in mice treated for 1 week with lipopolysaccharide (LPS) and polyinosinic-polycytidylic acid [Poly(I:C)]. C57BL6 mice (*n* = 6 per group) were i.p. injected every 2 days for 1 week with physiological water, LPS 10 µg, Poly(I:C) 200 µg, or LPS + Poly(I:C). Thymuses were analyzed **(A)** by flow cytometry for the percentage of B cells (CD19^+^ cells) or by PCR for CD19 **(B)**, CXCL13 **(C)**, CCL21 **(D)**, interferon (IFN)-β **(E)**, IFN-α **(F)**, and α-AChR **(G)** mRNA expression. Data were normalized on GAPDH. *p*-Values were assessed by the Mann–Whitney test and annotated as follows on graphs: *p < 0.05, **p < 0.01, and ***p < 0.001.

We next investigated if we could exacerbate Poly(I:C) with simultaneous LPS injections. Systemic administration of LPS is commonly used to study inflammation-associated behavioral changes in rodents. Moreover, LPS could also favor GC development (Table 1). After 1 week of injections, Poly(I:C) was able to increase the percentage of thymic B cells (Figures 3A,B) and thymic expression of CXCL13, CCL21, IFN-β, and α-AChR (Figures 3C–G), as previously demonstrated (18). We observed an increase in B cells in the thymus of LPS-treated mice by analyzing CD19 mRNA expression but not by flow cytometry (Figures 3A,B). We did not detect any effects of LPS by itself on the expression of CXCL13, CCL21, IFN-β, IFN-α, or α-ACHR mRNAs in the thymus (Figures 3C–G). However, LPS in combination with Poly(I:C) induced a huge increase in B cells in the thymus of mice as observed by flow cytometry and by PCR analyses (Figures 3A,B). The thymic recruitment of B cells in LPS/Poly(I:C)-injected mice was accompanied by a high overexpression of CXCL13 mRNA (Figure 3C) but not especially with changes in CCL21, IFN-β, or IFN-α mRNA expression compared to Poly(I:C)-injected mice (Figures 3D–G). We did not observe either a combined effect of Poly(I:C) and LPS on α-AChR mRNA expression (Figure 3F).

**Figure 4:**
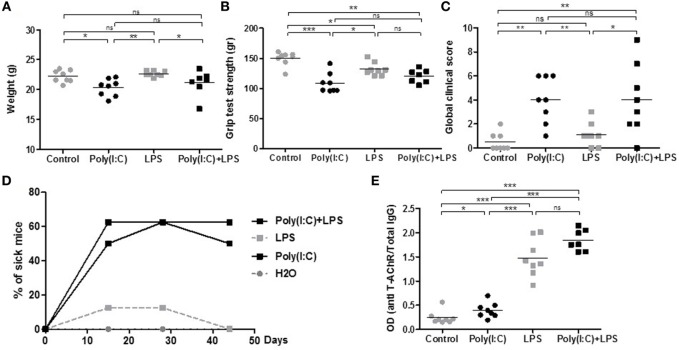
Clinical evaluation of mice treated for 6 weeks with lipopolysaccharide (LPS) and polyinosinic–polycytidylic acid [Poly(I:C)]. C57BL6 mice (*n* = 8 per group) were i.p. injected twice a week for 6 weeks with physiological water, LPS 10 µg, Poly(I:C) 200 µg, or LPS + Poly(I:C) (*n* = 7 as one mouse died at day 28). Clinical evaluations were done at different times and data obtained at day 44 are shown on graphs A–C and E. **(A)** Mice were weighted. **(B)** Muscle strength was measured with the grip test apparatus after exercise on a treadmill. **(C)** A global clinical score for each mouse was calculated taking into account the weight loss, the grip test, and the rotarod test. **(D)** The percentages of sick mice (with a global clinical score above 3) are shown in kinetic. **(E)** ELISA for anti-AChR antibodies. Data were normalized on the total level of IgGs. *p*-Values were assessed by the Mann–Whitney test and annotated as follows on graphs: *p < 0.05; **p < 0.01; and ***p < 0.001.

These effects indicated that LPS could exacerbate Poly(I:C) effects on thymic CXCL13 mRNA expression and favor a higher recruitment of B cells into the thymus after 1 week of injection. We thus investigated the effects of prolonged injections of LPS and Poly(I:C) for 6 weeks on MG symptoms appearance and thymic changes. In contrast to Poly(I:C) which decreased the animal weight, LPS had no effect (Figure 4A). Poly(I:C) and LPS separately were both able to decrease the mouse strength, but no exacerbated effect was observed when Poly(I:C) and LPS were injected together (Figure 4B). Using the global clinical test, we observed that symptoms were induced by Poly(I:C) and not really affected by LPS (Figures 4C,D). Analyzing circulating anti-AChR antibodies, we measured that Poly(I:C) induced anti-AChR production of around two times compared to controls. The effects of LPS were much pronounced with anti-AChR antibody productions around 6–7.5 times without or with Poly(I:C), respectively (Figure 4E). It is known that LPS can induce a global increase in IgG but here even once normalized on total IgGs, we observed that LPS induced a preferential production of anti-AChR antibodies. However, we did not observe associated MG symptoms when LPS was used on its own. Most likely, LPS induced a strong expression of anti T-AChR IgG1 antibodies that do not bind to complement and were not as pathogenic as IgG2 antibodies.

**Figure 5:**
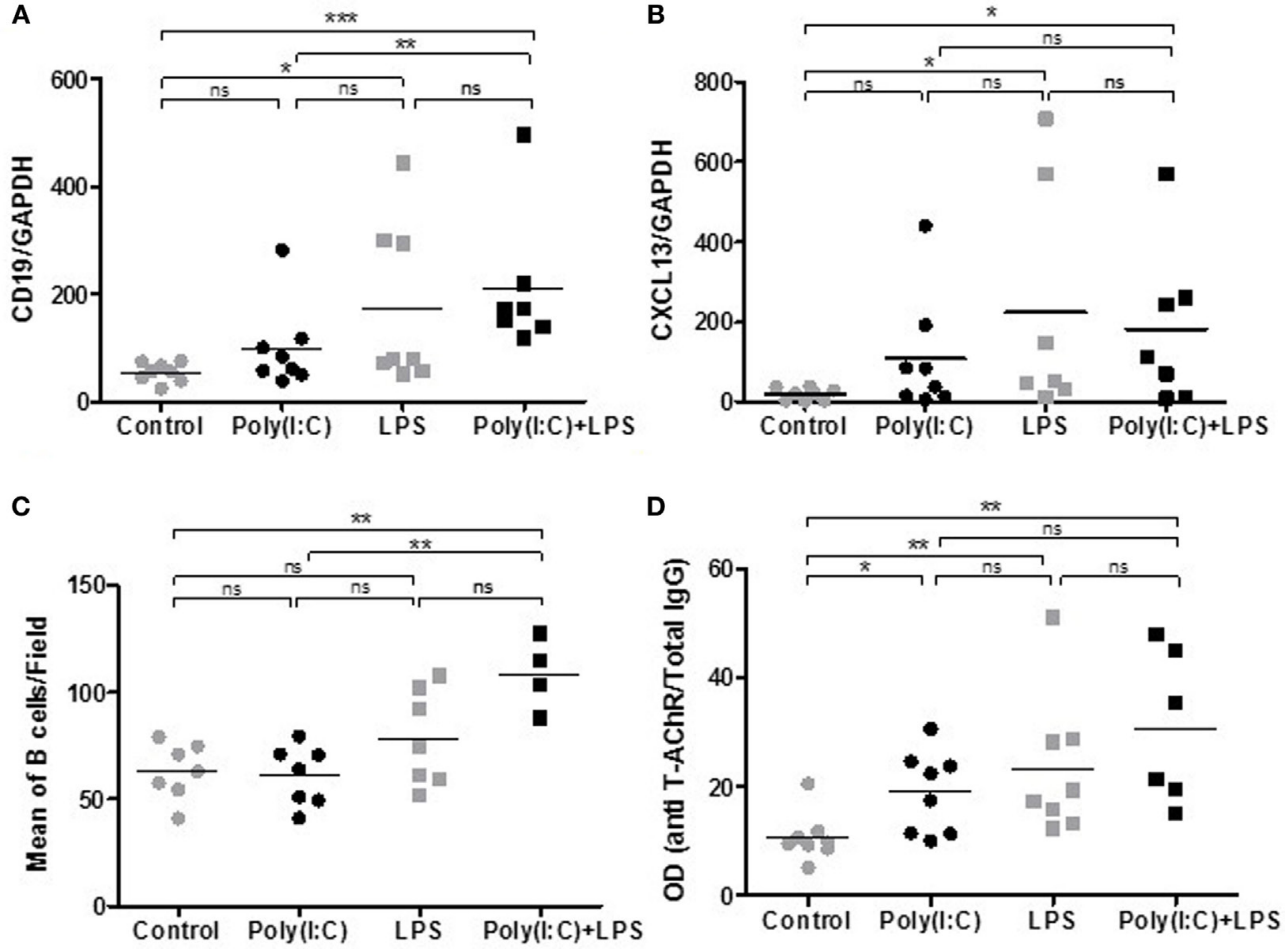

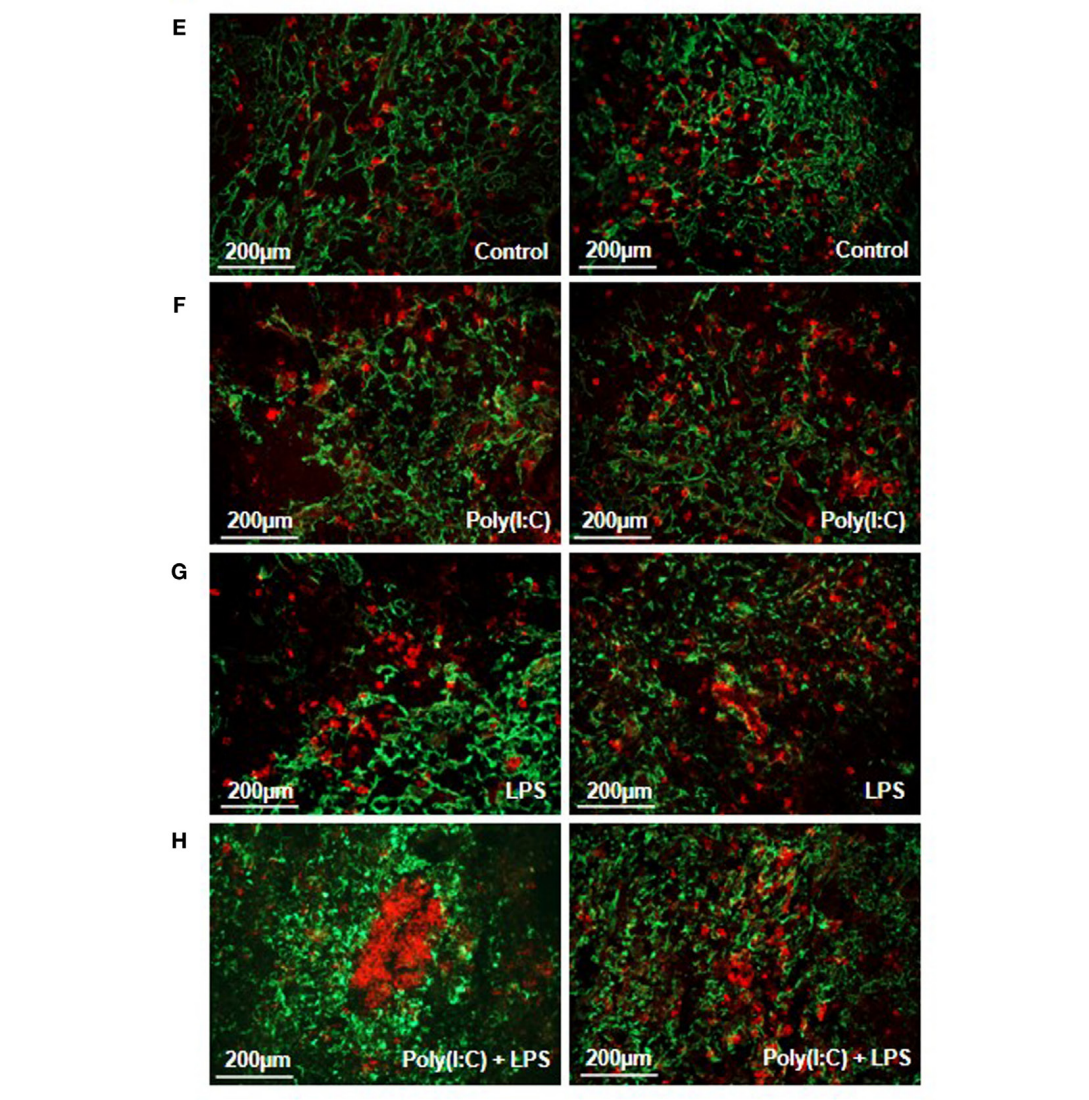
Analyses of thymic changes in mice treated for 6 weeks with lipopolysaccharide (LPS) and polyinosinic-polycytidylic acid [Poly(I:C)]. C57BL6 mice (*n* = 8 per group) were IP injected twice a week for 6 weeks with physiological water, LPS 10 µg, Poly (I:C) 200 µg, or LPS + Poly(I:C) (*n* = 7 as one mouse died at day 28). **(A,B)** PCR analyses for CD19 and CXCL13 mRNA. Data were normalized on GAPDH. **(D)** ELISA for anti-AChR antibodies on thymic extracts. Data were normalized on the total level of thymic IgGs. **(E–H)** Thymic sections were stained with an anti-K5-FITC antibody (green) and a biotinylated anti-B220 antibody plus a streptavidin Alexa-Fluor-594 (red). **(C)** The number of B cells was counted in 6–8 fields representative of thymic sections and each point correspond to the mean for each mice. In the Poly(I:C)/LPS group, two mice with B-cell clusters were excluded from the counting. **(E–H)** Representative thymic sections show in **(G)** the presence of B-cell clusters. *p*-Values were assessed by the Mann–Whitney test and annotated as follow on graphs: *p < 0.05; **p < 0.01; and ***p < 0.01.

We then analyzed if thymic changes were observed in mice at the end of the experiments. No significant differences were observed on thymic weights (data not shown). However, we measured an increase in expression of CD19 mRNA (Figure 5A), CD20 mRNA (data not shown), and CXCL13 mRNA (Figure 5B) in the thymus of mice injected with LPS or Poly(I:C)/LPS. In parallel, by immunohistochemistry, we observed that LPS but especially Poly(I:C)/LPS injections induced a significantly higher recruitment of B cells to the thymus as determined by cell counting on thymic sections (Figures 5C,E–H), and the presence of B-cell clusters was also observed in the thymus of a few mice as shown in Figure 5H. In parallel, analyzing the levels of anti-AChR antibodies in thymic fragments (Figure 5D), we observed significant increases in anti-AChR antibodies in all treated groups, as in the serum (Figure 4E). Even if within the group of Poly(I:C)/LPS treated mice, some of them displayed higher levels of anti-AChR antibodies, they did not especially correspond to thymic fragments for which lymphoid aggregates were observed by immunohistochemistry. This last observation could be due to the fact that proteins were extracted from different fragments. Altogether, these data suggest that thymic inflammation subsequent to LPS and Poly(I:C) signalization are important for tertiary lymphoid genesis in the thymus of mice.

## Discussion

As fully described in introduction, the MG thymus displays all the characteristics of TLOs. How the thymus acquired these features is not well-known but local inflammation seems to be mandatory. The overexpression in the MG thymus of IFN-β and IFN-I-induced genes suggests that MG could be triggered by pathogen infections. Inappropriate TLR signaling has been associated with various autoimmune diseases and more specifically persistent viral infection related to TLO development (18, 31, 32). Moreover, as detailed in Table 1, the activation of some TLR signaling pathway seems to play a central role in GC reactions. The overexpression of various TLRs has been observed in the MG thymus (24–26). Moreover, Poly(I:C) that activates TLR3 (but also other dsRNA-sensitive proteins) can induce in mice transient thymic changes recapitulating some characteristics of the MG thymus and lead to MG symptoms (18). Altogether, these data suggest that inappropriate TLR signaling activation might be responsible for ectopic GC development.

### Attempt to Induce Thymic Lymphoid Neogenesis in the Classical EAMG Model

In the classical EAMG model induced by immunizing mice with T-AChR, mice are quite resistant to develop MG symptoms despite the strong immune reaction triggered by the injections of T-AChR emulsified in CFA (27). In this model, mice develop antibodies against AChR associated with muscle weakness but this model does not recapitulate the human disease, as no tertiary lymphoid neogenesis is observed (28). Our objective was to develop a new EAMG model associated with thymic tertiary lymphoid neogenesis. The basic principle of experimental models for autoimmune diseases involves a strong inflammatory reaction associated with the use of CFA. CFA contains heat-inactivated MTB that could activate TLR2, TLR4, TLR9, and possibly TLR8 signaling pathways (33). However, it appears not sufficient to lead to thymic inflammation and GC development in the EAMG model.

As i.p. injections of Poly(I:C) can induce transient thymic changes (18), we challenged the EAMG model with concomitant Poly(I:C) injections. We observed that Poly(I:C) favored MG development as almost all mice had an elevated clinical score associated with MG symptoms. Unfortunately, even if Poly(I:C) exacerbated MG development in the EAMG model, we did not observe the induction of thymic changes associated with B-cell recruitment and ectopic GC development.

Lipopolysaccharide injections have also been used in the EAMG mouse model to try to substitute CFA as MTB can activate TLR4. C57BL/6 mice were immunized by several subcutaneous injections in foot-pads and shoulders with T-AChR and LPS emulsified in IFA or T-AChR emulsified in CFA. AChR-LPS/IFA-immunized mice develop clinical signs that are similar to those observed with the AChR/CFA immunization but B-cell activation leading to the production of the anti-AChR antibodies is different in AChR-LPS/IFA and AChR/CFA models. They demonstrated that CFA immunization requires CD4 costimulation of B cells to induce anti-AChR IgG2 secretion while LPS immunization does not. Unfortunately, in this study the ability of LPS to induce GC development in the thymus has not been investigated (34).

Despite the strong inflammatory effects of CFA, transduced whether or not by TLRs, it appears not sufficient to lead to thymic inflammation and GC development in the EAMG model even in association with Poly(I:C). This might be a consequence of the administration mode. Indeed, the injection of antigen-protein in CFA induces a local inflammation that might not efficiently impact the thymus.

### No Effects of TLR7 and TLR9 Agonists in Inducing or Sustaining Poly(I:C) Effects on Thymic B-Cell Recruitment

Regarding the early effects of Poly(I:C) on the mouse thymus and its late effects associated with anti-AChR antibodies production (18), our objective was to test the combined effects of Poly(I:C) with other TLR agonists: (1) to induce thymic tertiary neogenesis and (2) to develop a new MG mouse model that would be less stressful than the classical EAMG model using immunizations with CFA.

Only a few publications mentioned that TLR3 ligand enhances GC formation, as for example in the spleen during the tetanus toxoid vaccine response (35). In mice, TLR3 is weakly expressed on B-cell subsets compared to TLR1, TLR7, and TLR9, and it is especially detected on marginal zone B cells. Moreover, Poly(I:C) fails to stimulate B-cell proliferation (36). Consequently, Poly(I:C) might not be efficient enough to favor GC reactions. Except TLR3 that signals only through TRIF (TIR domain-containing adapter-inducing IFN-β), all TLRs interact with the intracellular adaptor myeloid differentiation primary response gene 88 (MyD88) to activate downstream signaling cascades. In mice, TLR signaling *via* MyD88 in B cells is supposed to enhance antibody response and favor GC development (37). It is also necessary to maintain long-lived plasma cells (38). We then used combined i.p. injections of Poly(I:C) with TLR7, TLR9, and TLR4 agonists (Imiquimod, CpG-C, and LPS, respectively), knowing that these TLR receptors are abnormally expressed in the thymus of MG patients (18, 24–26).

Imiquimod did not modify Poly(I:C) effects on the mouse thymus. This observation was very surprising as TLR7 signaling activation seems to be crucial for spontaneous GC development and plasmablast B-cell development (39–43). TLR7 is expressed on different B-cell subsets (36) and could regulate key checkpoints controlling GC development (44). Das et al. also recently demonstrated that TLR7 on follicular dendritic cells promotes autoreactive B-cell responses (45).

We have used CpG-C allowing both the production of IFN-α by plasmacytoid dendritic cells and B-cell stimulation. TLR9 activation in combination with Poly(I:C) was not effective either in sustaining Poly(I:C) effects on the mouse thymus. This observation was again very surprising as CpG is known to directly activate B cells and is one of the most potent B-cell mitogens known (46). *In vitro*, CpG stimulates plasma cell differentiation of naïve and memory B cells and can synergize with B-cell receptor (BCR) cross-linking to dramatically enhance B-cell proliferation (47). Rookhuizen and DeFranco have also demonstrated that TLR9 signaling acts in multiple complementary ways to increase the magnitude and enhance the quality of GC reactions (48). Moreover, a synergistic engagement of BCR and TLR9 signaling has been shown to effectively activate autoreactive B cells *in vitro* (49). However, TLR9 engagement could be more complex. In fact, the implication of TLR9 activation in mouse models has complex consequences as TLR9 on B cells restrains TLR7-mediated spontaneous autoimmunity in C57BL/6 mice (50). Moreover, repeated TLR9 activation has also been shown to disrupt GC formation (51).

High expression levels of TLR7 and TLR9 proteins have been observed in B cells and plasma cells of thymic GCs and lymphoid infiltrates in MG (25, 52). In the MG thymus, the expression of these TLRs colocalized with Epstein–Barr virus (EBV) antigens, and it has been suggested that EBV-associated TLR7 or TLR9 signaling may promote abnormal B-cell activation and proliferation leading to GC formations. As human is the only natural host of EBV infection, it can explain why we were not able to sustain Poly(I:C) effects on mouse thymus using TLR7 and TLR9 agonists.

### Combined Use of LPS and Poly(I:C) Induced Thymic B-Cell Recruitment

TLR4 is also overexpressed in MG thymuses (23, 24). Using LPS together with Poly(I:C) was very effective in potentiating some of the Poly(I:C) effects. After 1 week of coinjections, we observed an increased thymic recruitment of peripheral B cells associated with a higher expression of the B-cell chemokine CXCL13. A higher expression of CXCL13 has also been observed in mice upon LPS-induced acute endotoxin stress (53). This potentiating effect seems independent of IFN-I signaling as LPS did not induce IFN-β or IFN-α expression. We next investigated the effects of prolonged injections of LPS and Poly(I:C). LPS alone or LPS with Poly(I:C) did not have much effects on the clinical evaluations compared to controls or Poly(I:C) alone, respectively. However, surprisingly, we observed an increased expression of anti-AChR antibodies due to LPS but this increased expression did not induce more symptoms. In MG patients, there is no clear correlation between the anti-AChR titer and the severity of the disease, although such a correlation might be observed at the individual level.

Analyzing the thymic changes after prolonged coinjections of Poly(I:C) and LPS, we still observed a higher expression of CXCL13 mRNA associated with a higher proportion of thymic B cells and eventually the appearance of B-cell clusters in the thymus. In future experiments, markers should be used to investigate GC structure.

To further investigate lymphoid neogenesis in Poly(I:C)/LPS-stimulated mice, we also analyzed thymic sections for HEV development but did not detect HEVs (data not shown). HEVs are well known to allow the entrance of lymphocytes within SLOs and TLOs (1). However, HEVs are not always mandatory for cell entrance in peripheral tissues. For example, in the spleen that exceptionally does not possess HEVs, lymphocytes directly pass from the blood to the red pulp and afterward migrate to the white pulp. In the hyperplastic thymus of MG patients, even if HEVs are largely found, we also demonstrated the presence of CCL21 positive lymphatic vessels that could serve as afferent lymphatic vessels and allow the recruitment of peripheral B and T cells (8). In a transgenic mouse model with the specific overexpression of CCL21 in the thyroid, Martin et al. observed the development of HEVs in the thyroid of transgenic mice but they demonstrated that HEVs were not required for initial colonization of the thyroid by lymphocytes and even suggested that HEV formation may depend on the infiltrating cells and could be a later event on the formation of the lymphoid aggregates (54).

How LPS can sustain Poly(I:C) effects increasing B-cell recruitment associated with lymphoid aggregates? LPS has been known for long to be a potent polyclonal activator for mouse B cells (55). Its receptor TLR4 is expressed on all mouse B-cell subsets. However, at lower levels compared to TLR1, TLR7, and TLR9 (36). TLR4 stimulation increases B-cell polarization, migration, and directionality, especially in response to CXCL13 (56). Hwang et al. nicely showed that TLR4 signaling enhances B-cell trafficking into lymph nodes, induces B-cell clustering and interactions within lymph node follicles, leads to sustained B-cell proliferation, overcomes the restriction that limits the access of non-antigen activated B cells to GC dark zones, and enhances the generation of memory and plasma cells (57). Moreover, Garin et al. demonstrated that TLR4 is expressed on follicular dendritic cells and TLR4 plays an important role in their maturation and the proper development of GC reactions (58).

Poly(I:C) through thymic expression of IFN-β is able to induce the expression of MG autoantigen, the α-AChR, but also the higher expression of CXCL13 and CCL21, known to favor GC development (18). Our data suggest that LPS through its effects on CXCL13 and B-cells sustained Poly(I:C) effects favoring the higher recruitment of B cells and the development of B-lymphoid aggregates.

We previously observed using transgenic mice with thymic overexpression of CXCL13 that CXCL13 itself is not able to induce thymic B-cell-related changes associated with MG. We then demonstrated that inflammation is mandatory to reveal CXCL13 ability to recruit B cells and to induce B-lymphoid structures (29). Here, even if CXCL13 was highly expressed and inflammation triggered by LPS/Poly(I:C) injections, B-lymphoid structures were only observed in a few mice, and no HEVs were observed by immunohistochemistry. Here, the same observation was done as we only observed a few lymphoid aggregates. It is important to mention that in human not all MG patients display GCs either (4, 59). Moreover, we cannot exclude the fact that the thymus might be more refractory to TLO development in mice compared to human. In addition, the genetic background involved in human MG or other autoimmune diseases is not recapitulated in rodents. We can also set up the hypothesis that other factors might be needed for GC development. Lymphotoxin alpha or beta have been proposed to play a role but these molecules were not induced in the thymus of mice in our different experiments (data not shown).

## Conclusion

The implication of TLR agonists in autoimmune experimental models has been largely studied for their adjuvant effect or their direct effect in inducing or exacerbating these models. However, the potential role of TLR agonists in the development of ectopic lymphoid structures in organ-specific autoimmune diseases has been less investigated or maybe not observed. For example, in Sjögren’s syndrome, Nandula et al. demonstrated that repeated injections of Poly(I:C) to NZB/WF1 mice accelerate the development of salivary gland disease by causing a significant upregulation in chemokine expression and inflammatory cell infiltrations within the submandibular glands. However, the development of lymphoid structures was not described (60). In contrast, in MLR/Mp mice which spontaneously develop pancreatitis in the exocrine pancreatic tissues, Poly(I:C) accelerates the development of the disease and induce peripheral cell infiltration and lymphoid structure development in the pancreas (61). Regarding LPS, its direct implication in lymphoid neogenesis has also been punctually shown, as repeated intranasal administrations to neonatal C57BL/6 mice induces inducible bronchus-associated lymphoid tissue that displays TLO features (62).

Altogether these data demonstrated that tertiary lymphoid neogenesis is a complex process. In our experiments, even if thymic changes are induced by Poly(I:C) and sustained by LPS. Optimum GC reaction might be altogether species dependent, organ dependent, and/or TLR signaling pathway dependent. It has already been suggested that coactivation of extracellular and intracellular TLR by CpG and LPS may accelerate systemic autoimmune disease in the anti-dsDNA transgenic mice *in vivo* (63). Here, the efficacy of combined effects of Poly(I:C) and LPS also suggest that coinfection in individual could be at the base of particular inflammatory reactions leading to ectopic GC development associated with autoimmune diseases.

## Ethics Statement

Experiments on mice were approved by the local Ethics Committee (agreement no. 2569.01).

## Author Contributions

MR, BV, SM, and MAC performed and analyzed the experiments, read and revised the manuscript. SB-A read and revised the manuscript. RLP conceptualized the study, analyzed the results, and wrote the manuscript.

## Conflict of Interest Statement

The authors declare that the research was conducted in the absence of any commercial or financial relationships that could be construed as a potential conflict of interest.

## Abbreviations

AChR, acetylcholine receptor; CFA, complete Freund’s adjuvant; EAMG, experimental autoimmune myasthenia gravis; GC, germinal center; IFN, interferon; Ig, immunoglobulin; KO, knockout; LPS, lipopolysaccharide; MG, myasthenia gravis; poly(I:C), polyinosinic–polycytidylic acid; SLO, secondary lymphoid organ; TEC, thymic epithelial cell; TLO, tertiary lymphoid organ; TLR, toll-like receptor.
