# Supplementary material for: Use of Toll-Like Receptor Agonists to Induce Ectopic Lymphoid Structures in Myasthenia Gravis Mouse Models
Source: Front Immunol. 2017 Aug 25;8:1029. doi: 10.3389/fimmu.2017.01029 (PMC5609563; doi:10.3389/fimmu.2017.01029)
Supplement: Supplementary file 1 [file Data_Sheet_1.PDF]

Table S1 : List of primers for PCR

Genes	Mouse primers	
**α-AChR**	GTGCTGGGCTCTTTCATCTC	(f)
	TTCTGTGCGCGTTCTCATAC	(r)
**CCL21**	CCCTGGACCCAAGGCAGT	(f)
	AGGCTTAGAGTGCTTCCGGG	(r)
**CD19**	GGGACCTGGACTGTGACCTA	(f)
	AGGACAGCCAAAGTGTGGAG	(r)
**CXCL13**	TGAGGCTCAGCACAGCAA	(f)
	ATGGGCTTCCAGAATACCG	(r)
**GAPDH**	AACTTTGGCATTGTGGAAGG	(f)
	ACACATTGGGGGTAGGAACA	(r)
**IFN-α2**	TCTGTGCTTTCCTCGTGATG	(f)
	TTGAGCCTTCTGGATCTGCT	(r)
**IFN-β**	CCCTATGGAGATGACGGAGA	(f)
	CTGTCTGCTGGTGGAGTTCA	(r)
