# Supplementary material for: Use of Toll-Like Receptor Agonists to Induce Ectopic Lymphoid Structures in Myasthenia Gravis Mouse Models
Source: Front Immunol. 2017 Aug 25;8:1029. doi: 10.3389/fimmu.2017.01029 (PMC5609563; doi:10.3389/fimmu.2017.01029)
Supplement: Supplementary file 2 [file Data_Sheet_2.PDF]

Figure S1:

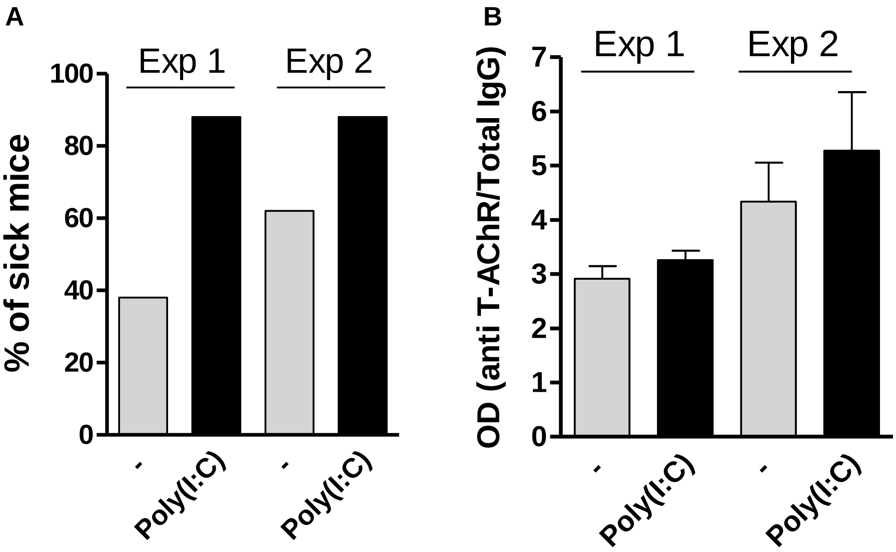

Figure 1S

Data from 2 independent experiments comparing the susceptibility of C57BL/6 mice immunized with T-AChR/CFA or T-AChR/CFA/Poly(I:C). Experiment 1 is detailed in Figure 1. In experiment 2, mice were only immunized twice. (A) Percentages of sick mice (with a global clinical score of at least 2) are shown 10-12 days after the last immunization. (B) ELISAs for anti-AChR antibodies were done on serum taken 10-12 weeks after the last immunization. For each experiment, data were normalized on a OD mean fixed at 0.02 for the control group of mice immunized only with CFA.
